# Research on the tripartite evolution strategy of prefabricated building promotion based on the deepening of demand-side interests

**DOI:** 10.1371/journal.pone.0290299

**Published:** 2023-09-14

**Authors:** Mengkai Liu, Yuyao Chen

**Affiliations:** School of Management, Wuhan University of Science and Technology, Wuhan, China; Swinburne University of Technology - Sarawak Campus, MALAYSIA

## Abstract

Under construction industry upgrading and environmental protection requirements, promoting prefabricated buildings is very important, but its development varies significantly in different regions. For example, it only accounts for 20% of the new construction area in China, and the overall development level is low. The promotion of prefabricated buildings involves multiple interests. Then, how to encourage all stakeholders to work together to promote its development is a crucial issue. This study obtains an evolutionary game model among the government, developers, and purchasers. Then the stability strategies of the stakeholders are given conditions. Finally, numerical simulation is used to validate theoretical findings and determine the sensitivity of key parameters to a subject’s behavior. The results show that: (1) There are two key factors that restrict the cooperation of stakeholders to promote prefabricated buildings, including the lack of government subsidies and the weak demand of the purchasers; (2) The government’s overall subsidy should not be too high, exceeding the government’s expenditure on environmental protection, and the subsidies for developers and purchasers must be reasonably allocated to effectively compensate for development benefits and purchase costs under limited expenditures. (3) Improving the residential environment of prefabricated buildings can significantly enhance the vested interests of purchasers, make up for their purchase costs, increase their willingness to purchase, and thus reduce the sales risk of developers.

## Introduction

Global environmental and ecological issues have gotten more attention recently as air pollution has increased. According to statistics, 50% of worldwide air pollution and 42% of global greenhouse gas emissions are caused by businesses associated with construction [1]. Some scholars have found that construction enterprises actively adopted green development behaviors and that migrating to industrial agglomerations was conducive to the sustainable development and environmental protection of construction enterprises [2]. From the current research, the benefits of using prefabricated buildings in construction projects for environmental protection, sustainable development, construction safety, and other factors have drawn attention from around the globe [3–5]. As can be seen, using prefabricated structures helps to advance the creation of ecological civilization and encourage the growth of "A green four sections." Prefabricated buildings have outstanding environmental and social benefits despite having a high construction cost. From the perspective of the whole life cycle of prefabricated buildings, prefabricated buildings have significant advantages in the positive impact of sustainable buildings and the role of protecting the environment [6–8]. Additionally, compared to traditional buildings, prefabricated buildings have the benefits of a quick construction time and lower labor requirements [9]. This is conducive to promoting the industrial upgrading of the construction industry, and at present, BIM, the Internet of Things, 3D printing, digital construction, and other technologies have also been widely used in prefabricated buildings [10–13]. This is not only conducive to improving the efficiency and quality of the construction industry but also further improving the environmental benefits of prefabricated buildings. Among them, digital green innovation management activities have become the core of low-carbon intelligent development for prefabricated construction enterprises to achieve sustainable urban development [14].

However, there are significant regional differences in prefabricated building development at the moment, as well as significant global variations in prefabricated building acceptance [15]. The development of prefabricated buildings is better in developed countries, according to the current state of development. For example, the market share of prefabricated buildings in the residential industry exceeds 80% in Sweden [16]. Due to urbanization, developing countries like China and India currently have a greater demand for prefabricated buildings [17]. However, prefabricated building development faces numerous challenges because the industrialization of construction in developing countries is frequently just getting started, the proportion of prefabricated buildings is frequently low, and the market is not yet mature. On the one hand, prefabricated buildings’ technical level still needs to be raised. The transportation of its components, the factors affecting the selection of the prefabricated component by manufacturers, and the planning of the prefabricated component supply chain under emergencies have all attracted the attention of researchers [18–20]. Some scholars have proposed measures to solve the defects of prefabricated components by reducing supply chain risk and improving component quality [21,22]. On the other hand, prefabricated buildings are currently facing a series of problems, such as cost [23]. Some scholars have proposed to reduce the cost of prefabricated buildings by optimizing module processing levels and supply chains [24,25].

It is necessary to take additional, effective steps to support the promotion of prefabricated buildings in order to address the aforementioned issues. This process is conducive to promoting the development of the prefabricated construction industry and related technological progress, and the cost of prefabricated buildings is affected by the scale effect to a certain extent, so it will be gradually alleviated in the promotion process of prefabricated buildings [26]. China, the greatest developing country in the world, released 389 prefabricated building-related policy papers between 2013 and 2017 to encourage the growth of prefabricated buildings [27]. In July 2020, the Ministry of Housing and Urban-Rural Development and seven other departments issued the ″Notice on Printing and Distributing Action Plans for the Creation of Green Buildings″, requiring an increase in the prefabrication rate. At present, supportive policies have achieved some results [28,29]. According to the data in the 2021 China Construction Annual Report, the newly started prefabricated construction area in the country reached 630 million square meters in 2020, accounting for about 20% of the newly increased construction area. However, this is still far from the State Council′s target of 30%. Additionally, prefabricated structures make up 91.7% and 40.2%, respectively, of the newly prefabricated construction areas in Shanghai and Beijing in 2020, which is much more than in other provinces and cities. There are still great differences in the development of prefabricated buildings in different regions [30]. According to this, the government has implemented numerous policies to promote prefabricated buildings and has seen some success. However, there are still some problems in the development of prefabricated buildings. Prefabricated buildings still make up a small part of all construction, and different regions have developed in quite diverse ways. From the perspective of policy focus, the policy pays relatively little attention to purchasers. However, encouraging the growth of the prefabricated industry chain involves more than just the cost of enterprises and government management; it also involves market demand and other elements [31]. It can be seen that the stakeholders in the development of prefabricated buildings have not been fully considered.

In summary, the promotion of prefabricated buildings can promote environmental protection and industrial upgrading. The government has launched many policies to promote the development of prefabricated buildings, which shows its strong willingness to promote prefabricated buildings. However, at present, the overall proportion of prefabricated buildings is still low, and the regional differences are large. The promotion effect has not yet reached expectations. Therefore, it is necessary to further study the limiting factors in the promotion of prefabricated buildings. By conducting a more thorough and in-depth investigation of the issues related to the development of prefabricated buildings, it can examine more effective promotion approaches.

## Literature review

Prefabricated buildings have been widely used worldwide, and the related problems they face have also received extensive attention from scholars worldwide. From the existing research, the development of prefabricated buildings is hindered by the joint influence of many parties. According to El-Abidi’s research results, the government and relevant practitioners still lack awareness of developing prefabricated buildings [32]. Therefore, this leads to some areas for improvement in the existing policies. Su et al. adopted text analysis to identify the relevant policies of prefabricated buildings and concluded that the pertinent current policies are sufficient but not balanced [33]. A comprehensive policy framework should pay attention to both developers’ willingness to build and purchasers’ demands [34]. At the same time, developers, as prominent participants in developing prefabricated buildings, have also received much attention. Based on online research, Rotimi found that the obstacles to developing prefabricated buildings are closely related to the professional skills shortage of relevant management, design, and residential construction practitioners [35]. Lee compared and analyzed the implementation conditions of modular buildings based on actual development cases and concluded that the construction cost was high [36]. NATALIE believed there are contradictions and obstacles between developers’ willingness to pursue short-term profits and the green development of prefabricated buildings [37]. Based on the developers’ perspective, Feldmann believes that industry attitude is the most critical factor affecting the development of modular buildings [38]. Therefore, how to protect the income of developers and promote their willingness to build is also a key factor. In addition to the issue of development revenue, Razkenari believed that off-site buildings also face significant resistance from the market environment [39]. Rosner adopted group experiments and found that people’s cognition, purchase cost, and policy factors affected people’s enthusiasm for choosing green buildings [40]. Rahimian made it clear that the public’s low acceptance of prefabricated buildings hindered its development [41]. There are differences in the research on prefabricated buildings in different regions, but there are still some common concerns. Gan surveyed the stakeholders of prefabricated buildings based on the fuzzy cognitive map. The results showed that all stakeholder groups mentioned policies, costs, and market demand [42]. Based on the above research on the development of prefabricated buildings, it can be seen that the promotion process mainly involves the interests of the government, developers, and purchasers.

Given the problem of multi-party interest balance, from the perspective of research methods, game theory is more mature in solving multi-agent problems. On the one hand, game theory is more common in promoting enterprises to adopt green, low-carbon technologies. Yin et al. discussed the stochastic differential game of low-carbon technology sharing in the collaborative innovation system of superior enterprises and inferior enterprises [43]. Wang et al. used evolutionary game theory to explore the impact of different environmental regulations on the diffusion of green technology innovations in manufacturing enterprises [44]. On the other hand, game theory is also widely used in the decision-making process in the construction industry. Guo et al. and Khanzadi et al., respectively, used game theory to establish the owner-supervision-contractor tripartite game mode and the owner-contractor two-party game model to explore the decision-making of all parties [45,46]. According to the above research, it is common to use game theory to study such problems and the decision-making of different subjects in the construction industry. It provides a theoretical framework for this paper’s application of evolutionary game theory to the investigation of prefabricated building promotion.

To promote the development of fabricated buildings, many Chinese scholars try to balance the interests of all parties to encourage its promotion effect. However, different scholars have different concerns about the subjects involved in promoting prefabricated buildings. Feng et al. believe that a good cooperation mechanism can effectively promote industrialization, so they take prefabricated manufacturers as the starting point and use evolutionary games to analyze their decision-making behavior so as to reduce the initial cost of the prefabricated building supply chain [47]. Chen et al. paid more attention to the game analysis among developers and discussed the economic compensation of prefabricated buildings in combination with the income-sharing model [48]. The above scholars discuss the behavior of different prefabricated manufacturers and construction developers, but they only study the game between similar subjects and lack a complete analysis of the whole promotion process. Among the research on different subjects, the current one that has attracted more attention is the game research based on the two subjects of ″government-developer″. Among them, Liu et al. combined prospect theory and evolutionary game theory analysis to find that the key to the long-term development of prefabricated buildings lies in reducing their construction costs [49]. Wang et al. and Chen et al. pointed out the impact of government regulation and management mechanisms on developers based on game theory [50,51]. It can be seen that although developers are the key subjects in choosing whether to develop prefabricated buildings or not, the government′s decision-making is also a key factor affecting the enthusiasm of developers. However, only considering the government’s and developers’ decision-making ignores the considerable pressure of the market environment developers face. The greater market risk will ultimately also seriously limit the enthusiasm of developers to choose prefabricated buildings. In the game analysis, some researchers considered the influence of purchasers in the promotion of prefabricated buildings [52,53]. Han et al. believed that purchasers′ preference for prefabricated buildings and cast-in-place buildings is also a key factor in developers′ decision-making, but they have not yet been possible to identify the specific factors that affect purchaser preferences [54]. But their research still fails to consider the balance of interests of the government, developers, and purchasers simultaneously, resulting in a lack of comprehensive analysis. This has led to good but not comprehensive problems in the relevant policies of prefabricated buildings, limiting their development. At present, only a small part of the research on the promotion and analysis of prefabricated buildings involves the three parties of government, developers, and purchasers [55–57]. But none of them has explicitly identified the impact of purchaser preferences on developers′ decisions. In addition, the parameters they chose for purchasers are excessively broad, and the advantages of buying prefabricated buildings are frequently summed up as incremental benefits. And they failed to conduct an in-depth analysis of the impact of purchaser decisions on developers and lacked more targeted conclusions. However, China′s prefabricated building policy lags behind its value realization, especially on the demand side [58]. Therefore, it is crucial to carefully consider the current situation of the purchasers in order to encourage the continued development of prefabricated buildings in developing countries. In addition, most studies fail to propose the specific scope of subsidies. Some literature calculates the scope of government subsidies from the perspectives of developers and purchasers without considering the government′s own profit and loss considerations. It can be seen that the analysis of government subsidies is not sufficient.

Based on the government’s high willingness to promote prefabricated buildings, the study builds a tripartite evolutionary game model of government-developers-purchasers and further analyzes the restrictions on the promotion of prefabricated buildings. And this study will put forward more refined suggestions on the balance of interests among the three parties.

## Research methods and ideas

### Evolutionary game theory

In evolutionary game theory, evolutionary stability strategy (ESS) and replication dynamics are two core concepts [59,60]. In the game process, based on bounded rationality, the game subject initially does not choose the optimal strategy. Evolutionary stability strategy (ESS) represents the process by which the subject tends to a certain stability strategy after repeated games. Replication dynamics represent a dynamic differential equation of the frequency at which a strategy is adopted by a population. The formula is as follows:

$$\frac{dx_{n}}{dt}=x_{n}[(U(n,s)‐U(s,s)]\;(n=1,2,\ldots ,k)$$ (1)

**Fig 1:**
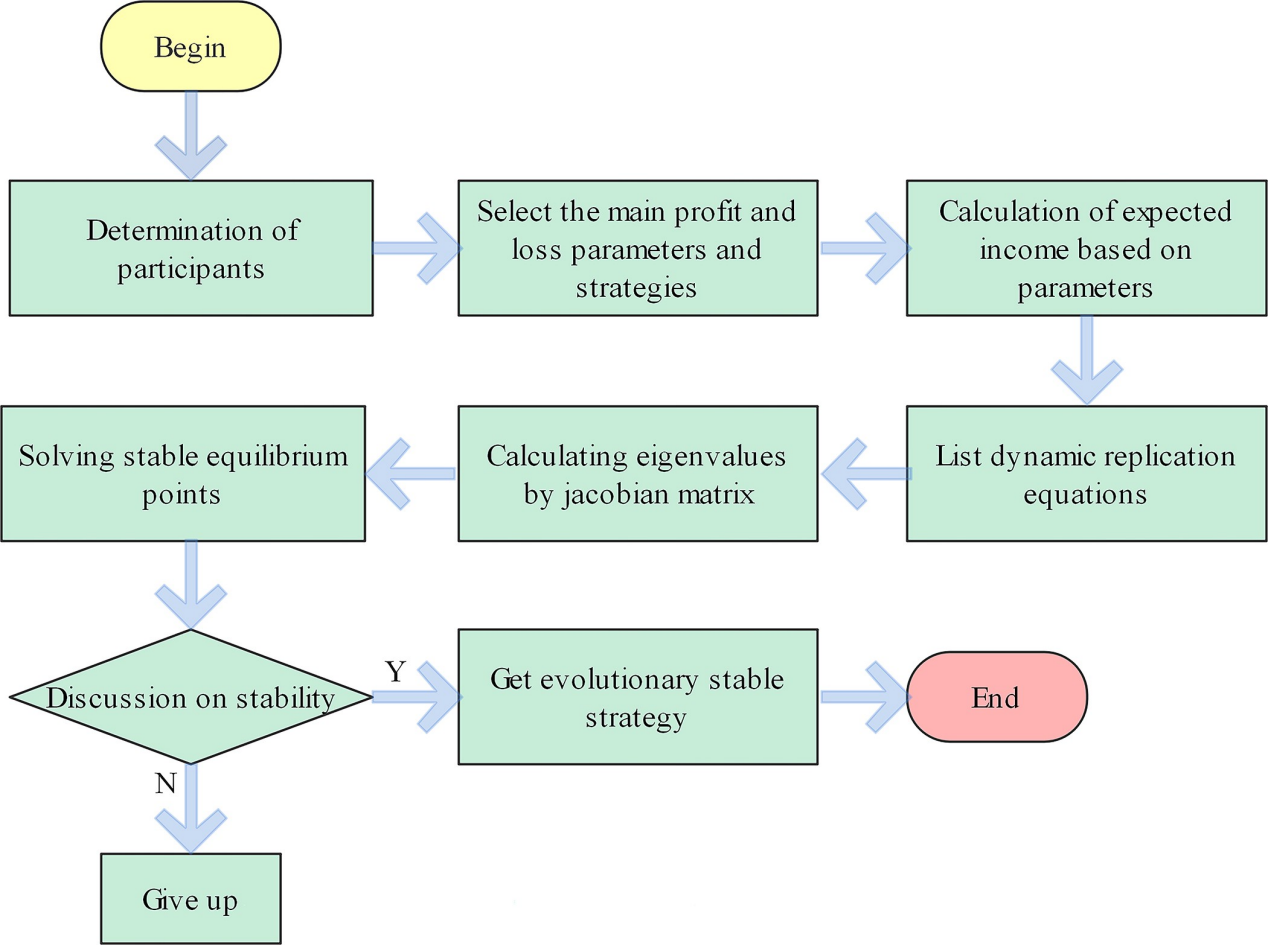
Evolutionary game theory process.

Where *x*_*n*_ represents the proportion or probability of adopting pure strategy *n* in a population, *U*(*n*, *s*) represents the fitness (expected return) of adopting strategy *n*, and *U*(*s*, *s*) represents the average fitness (average expected return). To solve ESS, the average fitness and strategy return need to be calculated first, and then they are substituted into the above formula to obtain the growth rate of the dynamic replication equation of a specific strategy. Finally, the eigenvalues of the corresponding strategy are calculated according to the Jacobian matrix, and the stability strategy is judged according to the eigenvalues. The calculation process is shown in Fig 1.

### Research route

The research object and purpose are determined through background research and literature reviews.

The behavior strategy combination among the game subjects is divided based on the relationship and influence between the government, developers, and purchasers. Combined with the current research, the profit and loss parameters of the three parties are selected to establish the tripartite evolutionary game model and calculate the corresponding income loss of each subject when adopting different strategies. Then, according to the dynamic replication equation, the eigenvalues of each strategy combination are calculated to judge the stability of different strategies, and the conditions required to reach the stable state are analyzed.

The influence of key parameters on the steady-state evolution process is analyzed by simulation, and the key parameters of the government, developers, and purchasers are simulated and compared.

Based on the above game analysis results and the sensitivity of parameters in the simulation experiment, the discussion and conclusions for the promotion of prefabricated buildings are given.

**Fig 2:**
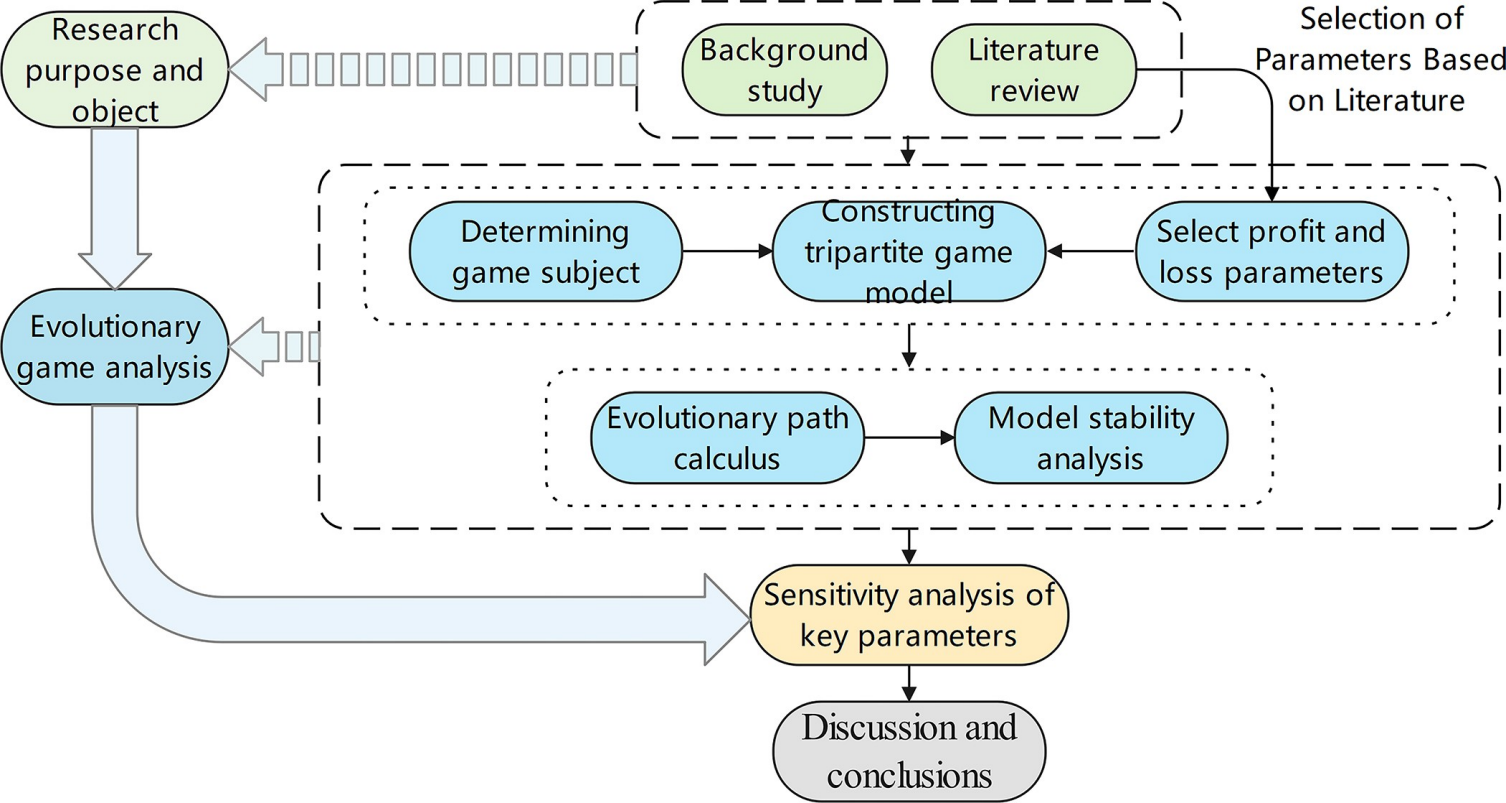
Technology roadmap.

The technology roadmap is shown in Fig 2.

## Evolutionary game analysis of prefabricated buildings promotion

### Evolutionary game model construction

At present, many scholars have adopted evolutionary game models to solve related problems. The tripartite evolutionary game model for the promotion of prefabricated buildings in this study is based on a comparative analysis of the modeling process in the above literature. The model-building procedure is as follows:

(1) Game-subject analysis

**Fig 3:**
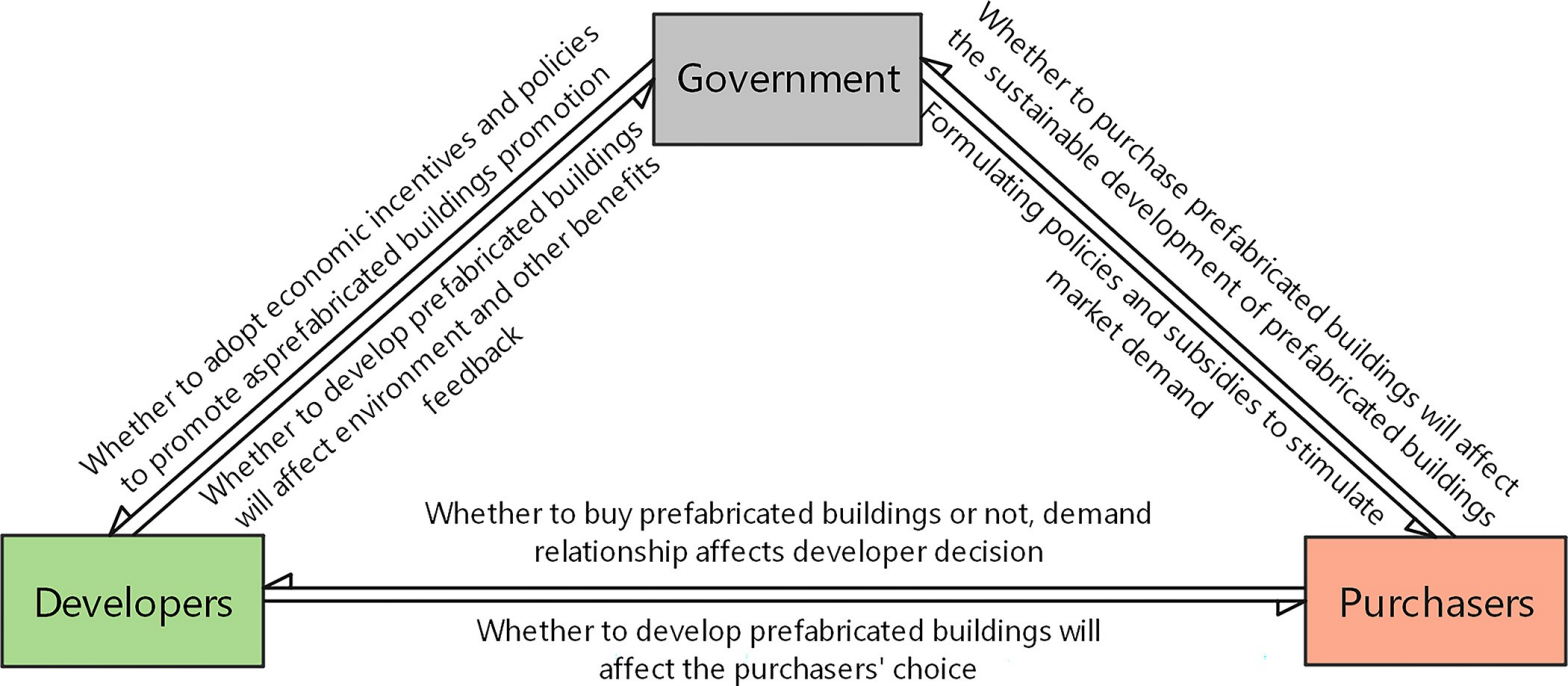
Schematic diagram of evolution subject relationship.

The government, developers, and purchasers are selected as the game subjects. The evolutionary game is based on the assumption of bounded rationality. The three subjects constantly change their strategies to achieve the optimal strategy in the game process. The interaction among the three is shown in Fig 3.

According to the relationship between the three parties involved in the promotion of prefabricated buildings, it can be seen that the purpose of the government is to promote environmental protection and the upgrading of the construction industry, so its willingness to promote prefabricated buildings is strong. Its main behavior is to encourage developers and purchasers to choose prefabricated buildings. The government harvests environmental, social, and economic benefits through the promotion of prefabricated buildings. Developers demand enough profit. The choice of prefabricated buildings by developers is influenced by their personal interests, the promotion of government incentives, and the supply and demand dynamics in the purchaser market. The purchasers demand to buy comfortable and economical housing; their choice is subject to government policies and subsidy incentives and developers′ supply constraints.

**Fig 4:**
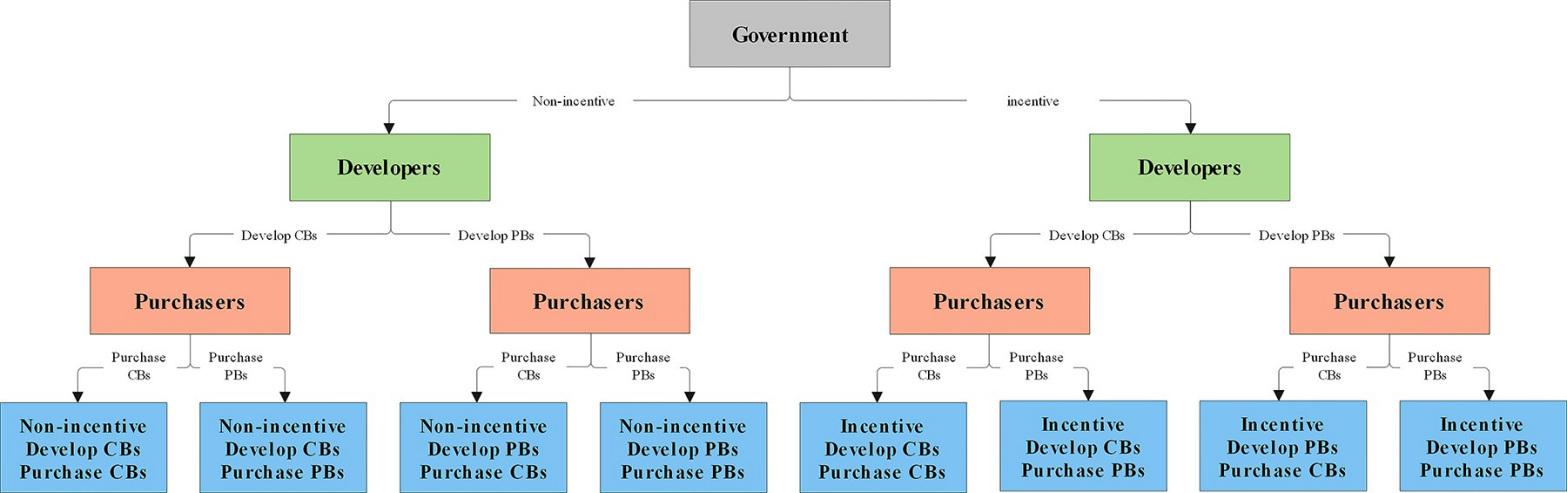
Tripartite behavior strategy combination.

The government′s strategy is to adopt economic incentives or not, the developers′ strategy is to develop fabricated buildings or cast-in-place buildings, and the purchasers′ strategy is to purchase fabricated buildings or cast-in-place buildings. The three parties′ evolutionary game model is established, and the evolutionary game strategy tree is shown in Fig 4. In Fig 4, PBs are used to refer to prefabricated buildings, and CBs are used to refer to cast-in-place buildings.

(2) Decision probability hypothesis

The probability that the government will adopt economic incentives is *x*, and the probability that the government does not adopt economic incentives is 1 − *x*; The probability that developers choose prefabricated buildings is *y*, and the probability of choosing cast-in-place buildings is 1-*y*; The probability of the purchasers choosing prefabricated buildings is *z*, and the probability of choosing cast-in-place buildings is 1-*z*.

(3) Model hypothesis

Evolutionary game parameter assumptions are shown in Table 1. In the process of building models, the selection of indicators should be based on the above literature [61]. Among them, the selection of parameters in this paper is based on the development of prefabricated buildings, combined with the above literature induction. In addition, a new parameter relating to the vested interests of purchasers is added to further deepen the analysis of the purchasers′ interests.

**Table 1:** Parameter assumption table of evolutionary game.

Game subject	Parameter settings and assumptions (Parameter values are positive)	References
Government	*G*_*1*_: The cost of policymaking when the government implements fabricated buildings.	[51,53]
	*G*_*2*_: Developers develop prefabricated buildings, providing additional social, economic, and environmental benefits to the government, and additional benefits for the future recovery and demolition of prefabricated buildings.	[50–53,56,57]
	*G*_*3*_: The government′s economic incentive costs, such as subsidies, tax relief, and incentives for prefabricated buildings. Among them, the proportion of subsidies received by the purchasers in total subsidy amount is *r*.	[50–53,57]
	*G*_*4*_: Environmental control costs incurred by the government without incentives to implement prefabricated buildings.	[51,52]
Developers	*D*_*1*_: When developers meet the prefabrication rate, they obtain economic subsidies and rewards from the government (including financial support and policy support), where the economic incentive is (1−*r*)*G*_*3*_.	[56,57]
	*D*_*2*_: The risk cost of sales loss caused by purchasers′ concerns about prefabricated buildings.	[54,58]
	*D*_*3*_: The direct incremental construction cost of prefabricated buildings developed by developers.	[51,53,56,57]
	*D*_*4*_: Developers′ benefit from reducing the construction period by choosing prefabricated buildings compared with cast-in-place buildings.	[9]
	*D*_*5*_: Social benefits and corporate reputation benefits of developers choosing prefabricated buildings	[53,56,57]
	*D*_*6*_: The incremental cost of environmental protection measures for developers choosing cast-in-place buildings compared with prefabricated buildings.	[3,6–8]
Purchasers	*O*_*1*_: The increased cost of purchasing prefabricated buildings compared with ordinary buildings.	[56,57]
	*O*_*2*_: The policy benefits obtained by the purchasers when buying prefabricated buildings include increasing the amount of purchaser accumulation funds and giving priority to loans.	[56]
	*O*_*3*_: Residential environmental benefits are obtained by purchasing prefabricated buildings.	The interest of the purchasers is an important factor in improving the acceptance of prefabricated buildings. The residential environment is the content of vested interests that purchasers care about.

According to the hypothetical data and the above strategy combination, the tripartite evolutionary game matrix is established. The calculation process is as follows: When Strategy 1 is adopted, because the government does not encourage it, there is no subsidy expenditure *G*_*3*_. Developers choose cast-in-place buildings, so there is no income *G*_*2*_, and the government needs to face the cost *G*_*4*_ and fixed expenditure *G*_*1*_ generated by non-incentives. Therefore, the government′s income is −*G*_*1*_–*G*_*4*_. Other calculation results are shown in Table 2. In Table 2, PBs are used to refer to prefabricated buildings, and CBs are used to refer to cast-in-place buildings.

**Table 2:** Tripartite profit and loss matrix.

Strategy ranking		Government	Developer	Purchaser
Strategy 1	(Non-incentive, Develop CBs, Purchase CBs)	-*G*_*1*_-*G*_*4*_	-*D*_*6*_	0
Strategy 2	(Non-incentive, Develop CBs, Purchase PBs)	-*G*_*1*_-*G*_*4*_	-*D*_*6*_	*-O*_*1*_*+O*_*2*_+*O*_*3*_
Strategy 3	(Non-incentive, Develop PBs, Purchase CBs)	-*G*_*1*_-*G*_*4*_+*G*_*2*_	*D*_*1*_-(1-*r*)*G*_*3*_-*D*_*2*_-*D*_*3*_+*D*_*4*_+*D*_*5*_	0
Strategy 4	(Non-incentive, Develop PBs, Purchase PBs)	-*G*_*1*_-*G*_*4*_+*G*_*2*_	*D*_*1*_-(1-*r*)*G*_*3*_-*D*_*2*_-*D*_*3*_+*D*_*4*_+*D*_*5*_	*-O*_*1*_*+O*_*2*_+*O*_*3*_
Strategy 5	(Incentive, Develop CBs, Purchase CBs)	-*G*_*1*_	-*D*_*6*_	0
Strategy 6	(Incentive, Develop CBs, Purchase PBs)	-*G*_*1*_-*rG*_*3*_	-*D*_*6*_	*-O*_*1*_*+O*_*2*_+*O*_*3*_*+rG*_*3*_
Strategy 7	(Incentive, Develop PBs, Purchase CBs)	-*G*_*1*_+*G*_*2*_-(1-*r*)*G*_*3*_	*D*_*1*_-*D*_*2*_-*D*_*3*_+*D*_*4*_+*D*_*5*_	0
Strategy 8	(Incentive, Develop PBs, Purchase PBs)	-*G*_*1*_+*G*_*2*_-*G*_*3*_	*D*_*1*_-*D*_*2*_-*D*_*3*_+*D*_*4*_+*D*_*5*_	*-O*_*1*_*+O*_*2*_+*O*_*3*_*+rG*_*3*_

### Evolution path of the evolutionary game model

The expected returns *U*_*g*1_ and *U*_*g*2_ of the government′s choice of incentives and non-incentives are as follows:

$$U_{g1}=yz(-G_{1}+G_{2}-G_{3})+y(1-z)(-G_{1}+G_{2}-(1-r)G_{3})-(1-y)z(G_{1}+rG_{3})-(1-y)(1-z)G_{1}$$ (2)

$$U_{g2}=yz(-G_{1}+G_{2}-G_{4})+y(1-z)(-G_{1}+G_{2}-G_{4})-(1-y)z(G_{1}+G_{4})-(1-y)(1-z)(G_{1}+G_{4})=y(-G_{1}+G_{2}-G_{4})-(1-y)(G_{1}+G_{4})$$ (3)

The dynamic replication equation of government is as follows:

$$F_{\left(x\right)}=\frac{dx}{dt}=x(1-x)(U_{g1}-U_{g2})=x(1-x)[(-y(z+(1-z)(1-r))+(1-y)zr)G_{3}+G_{4}]=x(1-x)[-yG_{3}+y(1-z)rG_{3}-(1-y)zrG_{3}+G_{4}]$$ (4)

The expected returns *U*_*d*1_ and *U*_*d*2_ that developers choose to develop prefabricated buildings and cast-in-place buildings are as follows:

$$U_{d1}=xz(D_{1}-D_{2}-D_{3}+D_{4}+D_{5})+x(1-z)(D_{1}-D_{2}-D_{3}+D_{4}+D_{5})+(1-x)z[D_{1}-D_{2}-D_{3}-(1-r)G_{3}+D_{4}+D_{5}]+(1-x)(1-z)[D_{1}-D_{2}-D_{3}-(1-r)G_{3}+D_{4}+D_{5}]=D_{1}-D_{2}-D_{3}-(1-x)(1-r)G_{3}+D_{4}+D_{5}$$ (5)

$$U_{d2}=xz({-D}_{6})+x(1-z)({-D}_{6})+(1-x)z({-D}_{6})+(1-x)(1-z)({-D}_{6})=-D_{6}$$ (6)

The dynamic replication equation of the developers is as follows:

$$F_{\left(y\right)}=\frac{dy}{dt}=y(1-y)(U_{d1}-U_{d2})=y(1-y)[D_{1}+D_{4}+D_{5}+D_{6}-D_{2}-D_{3}-(1-x)(1-r)G_{3}]$$ (7)

The expected returns *U*_*o*1_ and *U*_*o*2_ of purchaser choosing to purchase prefabricated buildings and cast-in-place buildings are as follows:

$$U_{o1}=xy(-O_{1}+O_{2}+O_{3}+rG_{3})+x(1-y)(-O_{1}+O_{2}+O_{3}+rG_{3})+(1-x)y(-O_{1}+O_{2}+O_{3})+(1-x)(1-y)(-O_{1}+O_{2}+O_{3})=O_{2}+O_{3}+xrG_{3}-O_{1}$$ (8)

$$U_{o2}=0$$ (9)

The dynamic replication equation of the purchasers is as follows:

$$F_{\left(z\right)}=\frac{dz}{dt}=z(1-z)(U_{o1}-U_{o2})=z(1-z)(O_{2}+O_{3}+xrG_{3}-O_{1})$$ (10)

The above Formulas (4), (7), and (10) are combined to obtain the Jacobian matrix of the tripartite game model. The calculation formula is as follows:

$$J=[\begin{array}{ccc} \frac{\partial F_{\left(x\right)}}{\partial x} & \frac{\partial F_{\left(x\right)}}{\partial y} & \frac{\partial F_{\left(x\right)}}{\partial z}\\ \frac{\partial F_{\left(y\right)}}{\partial x} & \frac{\partial F_{\left(y\right)}}{\partial y} & \frac{\partial F_{\left(y\right)}}{\partial z}\\ \frac{\partial F_{\left(z\right)}}{\partial x} & \frac{\partial F_{\left(z\right)}}{\partial y} & \frac{\partial F_{\left(z\right)}}{\partial z} \end{array}]=[\begin{array}{ccc} J_{11} & J_{12} & J_{13}\\ J_{21} & J_{22} & J_{23}\\ J_{31} & J_{32} & J_{33} \end{array}]$$ (11)

The above equation is brought into the Jacobian matrix, and the calculation results are shown in Table 3.

**Table 3:** Jacobian matrix.

(1-2*x*)[-*yG*_*3*_+*y*(1-*z*)*rG*_*3*_-(1-*y*)*zrG*_*3*_+*G*_*4*_]	*x*(1-*x*)[-*G*_*3*_+(1-*z*)*rG*_*3*_+*zrG*_*3*_]	*x*(1-*x*)[-*yrG*_*3*_-(1-*y*)*rG*_*3*_]
*y*(1-*y*)(1-*r*)*G*_*3*_	(1-2*y*)[*D*_*1*_-*D*_*2*_-*D*_*3*_+*D*_*4*_+*D*_*5*_+*D*_*6*_-(1-*x*)(1-*r*)*G*_*3*_]	0
*z(*1*-z)rG*_*3*_	0	*(*1-2*z) (O*_*2*_+*O*_*3*_*+xrG*_*3*_*-O*_*1*_*)*

In view of the fact that the asymptotic stable solution of the dynamic replication system of a multi-population evolutionary game must be a strict Nash equilibrium solution, only the following eight equilibrium points are considered: E_1_(0,0,0)、E_2_(0,0,1)、E_3_(1,0,0)、E_4_(0,1,0)、E_5_(0,1,1)、E_6_(1,0,0)、E_7_(1,1,0)、E_8_(1,1,1). The above equilibrium points are introduced into the Jacobian matrix. The eigenvalues of the above equilibrium points are shown in Table 4. The equilibrium strategy is stable only when all eigenvalues are negative.

**Table 4:** Balance point judgment table.

Point of equilibrium	*λ*_*1*_	*λ*_*2*_	*λ*_*3*_	Asymptotic stability
E_1_(0,0,0)	*G*_*4*_	*D*_*1*_-*D*_*2*_-*D*_*3*_+*D*_*4*_+*D*_*5*_+*D*_*6*_-(1-*r*)*G*_*3*_	*-O*_*1*_ *+O*_*2*_ *+O*_*3*_	Unstable
E_2_(0,0,1)	-*rG*_*3*_+*G*_*4*_	*D*_*1*_-*D*_*2*_-*D*_*3*_+*D*_*4*_+*D*_*5*_+*D*_*6*_-(1-*r*)*G*_*3*_	*O*_*1*_ *-O*_*2*_ *-O*_*3*_	Uncertain
E_3_(0,1,0)	(*r*-1)*G*_*3*_+*G*_*4*_	-*D*_*1*_+*D*_*2*_+*D*_*3*_-*D*_*4*_-*D*_*5*_-*D*_*6*_+(1-*r*)*G*_*3*_	*-O*_*1*_ *+O*_*2*_ *+O*_*3*_	Uncertain
E_4_(0,1,1)	-*G*_*3*_+*G*_*4*_	-*D*_*1*_+*D*_*2*_+*D*_*3*_-*D*_*4*_-*D*_*5*_-*D*_*6*_+(1-*r*)*G*_*3*_	*O*_*1*_ *-O*_*2*_ *-O*_*3*_	Uncertain
E_5_(1,0,0)	-*G*_*4*_	*D*_*1*_-*D*_*2*_-*D*_*3*_+*D*_*4*_+*D*_*5*_+*D*_*6*_	*-O*_*1*_ *+O*_*2*_ *+O*_*3*_ *+rG*_*3*_	Uncertain
E_6_(1,0,1)	*rG*_*3*_-*G*_*4*_	*D*_*1*_-*D*_*2*_-*D*_*3*_+*D*_*4*_+*D*_*5*_+*D*_*6*_	*O*_*1*_ *-O*_*2*_ *-O*_*3*_ *-rG*_*3*_	Uncertain
E_7_(1,1,0)	(1-*r*)*G*_*3*_-*G*_*4*_	-*D*_*1*_+*D*_*2*_+*D*_*3*_-*D*_*4*_-*D*_*5*_-*D*_*6*_	*-O*_*1*_ *+O*_*2*_ *+O*_*3*_ *+rG*_*3*_	Uncertain
E_8_(1,1,1)	*G*_*3*_-*G*_*4*_	-*D*_*1*_+*D*_*2*_+*D*_*3*_-*D*_*4*_-*D*_*5*_-*D*_*6*_	*O*_*1*_ *-O*_*2*_ *-O*_*3*_ *-rG*_*3*_	Uncertain

### Stability analysis of government, developers, and the purchasers′ tripartite strategy

The mixed strategy of the government, developers, and purchasers influences each other when it reaches a stable state, so it is necessary to consider the internal relations among the three. On the one hand, government subsidies have a significant impact on the incremental cost of prefabricated buildings. Li et al. (2013) took the residential sample as an example. It was concluded that the fixed cost of prefabricated buildings was high and could not be reduced in the short term [62]. If the government does not take economic subsidies, the incremental cost of developing prefabricated buildings in a certain period is at a large value. Luo et al. (2020) adopted the FCM model to analyze the factors affecting prefabricated buildings′ cost and believed that the fundamental reason was the scale effect [26]. Based on the above literature, if the government does not provide certain economic incentives in the early stages for developers, the incremental cost of prefabricated buildings is often higher than the benefits it brings. This results in a consistently small prefabricated building market, so the cost is always difficult to decline. On the one hand, there is a certain sales risk for developers to develop prefabricated buildings. When purchasers′ willingness to purchase prefabricated buildings is low, developers face greater losses due to problems such as capital turnover and repayment due to sales risks. Accordingly, it is thought that developers will tend to choose prefabricated buildings only when the incremental cost of prefabricated buildings and sales risks are both low. This is to further ensure the careful consideration of the interests of the three and deepen the analysis of the vested interests of the purchasers.

Combined with the above equilibrium point judgment table, *r* is not infinitely small. When the government′s subsidy expenditure for promoting prefabricated buildings exceeds the cost of environmental governance without promoting prefabricated buildings, namely, *G*_*3*_>*G*_*4*_. At this time, only the eigenvalues λ_1_<0 of the equilibrium points E_2_, E_3_, E_4_, and E_5_ may be established. However, the equilibrium point E_5_ means that the subsidy of government expenditure at this time has exceeded the environmental governance cost of not promoting prefabricated buildings. It is inconsistent with its actual situation, so this situation is excluded. For developers, if the government does not take subsidies, the incremental cost of developing prefabricated buildings at this time is higher, so *D*_*1*_+*D*_*4*_+*D*_*5*_+*D*_*6*_<*D*_*2*_+D_3_. In the equilibrium point where the above eigenvalues λ_1_<0 may hold, only the eigenvalue λ_2_<0 of the equilibrium point E_2_. Based on this situation, consider the stability of the purchasers. Only when the purchasers′ policy and residential environmental benefits from purchasing prefabricated buildings are higher than the incremental cost of purchasing prefabricated buildings, that is, to meet condition 1: *rG*_*3*_*>G*_*4*_, *D*_*1*_*+D*_*4*_*+D*_*5*_*+D*_*6*_*<D*_*2*_*+D*_*3*_, *O*_*1*_<*O*_*2*_+*O*_*3*_. The government, developers, and purchasers will eventually evolve to point E_2_(0,0,1). That is, the evolution strategy is (Non-incentive, Develop cast-in-place buildings, Purchase prefabricated buildings).

Conversely, when the government′s subsidy expenditure for implementing prefabricated buildings is lower than the environmental governance cost of not implementing prefabricated buildings, namely, *G*_*3*_<*G*_*4*_. At this time, only the eigenvalues λ_1_ < 0 of the equilibrium points E_5_, E_6_, E_7_, and E_8_ are established. The government tends to take economic subsidies when developers get subsidies after the incremental cost is low, *D*_*1*_*-D*_*2*_*-D*_*3*_*+D*_*4*_*+D*_*5*_*+D*_*6*_ difference positive and negative depends on sales risk. Based on this situation, the influence of the purchasers on the demand for prefabricated buildings is further considered. If the policy and residential environmental benefits of purchasers buying prefabricated buildings are low and it is impossible to compensate for the additional purchase cost, namely, *O*_*1*_>*O*_*2*_+*O*_*3*_+*rG*_*3*_. In the equilibrium point where the above eigenvalues λ_1_<0 may hold, only the eigenvalues λ_3_<0 of the equilibrium points E_5_ and E_7_ hold. The purchasers tend not to buy prefabricated buildings, and the sales risk of developers choosing to develop prefabricated buildings is high. At this time, only the eigenvalue λ_2_ < 0 of the equilibrium point E_5_ is established. Therefore, when condition 2 is satisfied: *G*_*3*_<*G*_*4*_, *D*_*1*_*+D*_*4*_*+D*_*5*_*+D*_*6*_*<D*_*2*_*+D*_*3*_, *O*_*1*_>*O*_*2*_+*O*_*3*_+*rG*_*3*_, the government, developers and the purchasers will eventually evolve to point E_5_(1,0,0), that is, the evolution strategy is (Incentive, Develop cast-in-place buildings, Purchase cast-in-place buildings); If the purchasers′ profit from buying prefabricated buildings covers the incremental purchase cost, namely, *O*_*1*_<*O*_*2*_+*O*_*3*_+*rG*_*3*_. In the equilibrium point where the above eigenvalues λ_1_<0 may hold, only the eigenvalues λ_3_<0 of the equilibrium points E_6_ and E_8_ hold. The purchasers have a strong willingness to buy prefabricated buildings. At this time, the sales risk of developing prefabricated buildings is low. At this time, only the eigenvalue λ_2_ < 0 of the equilibrium point E_8_ is established. Therefore, when condition 3 is satisfied: *G*_*3*_<*G*_*4*_, *D*_*1*_*+D*_*4*_*+D*_*5*_*+D*_*6*_*>D*_*2*_*+D*_*3*_, *O*_*1*_<*O*_*2*_+*O*_*3*_+ *rG*_*3*_. The government, developers, and purchasers will eventually evolve to point E_8_(1,1,1). That is, the evolution strategy is (Incentive, Develop prefabricated buildings, Purchase prefabricated buildings). The stability of the equilibrium point and its conditions are shown in Table 5.

**Table 5:** Stability conditions of equilibrium points.

Point of equilibrium	Eigenvalue	Stability condition
E_2_(0,0,1)	λ_1_ = *-rG*_*3*_*+G*_*4*_	Condition 1: *rG*_*3*_*>G*_*4*_、*D*_*1*_*+D*_*4*_*+D*_*5*_*+D*_*6*_*<D*_*2*_*+D*_*3*_、*O*_*1*_<*O*_*2*_+*O*_*3*_
	λ_2_ = *D*_*1*_*-D*_*2*_*-D*_*3*_*+D*_*4*_*+D*_*5*_*+D*_*6*_*-(*1*-r)G*_*3*_	
	λ_3_ = *O*_*1*_*-O*_*2*_*-O*_*3*_	
E_5_(1,0,0)	λ_1_ = *-G*_*4*_	Condition 2: *G*_*3*_<*G*_*4*_、*D*_*1*_*+D*_*4*_*+D*_*5*_*+D*_*6*_*<D*_*2*_*+D*_*3*_、*O*_*1*_>*O*_*2*_+*O*_*3*_+ *rG*_*3*_
	λ_2_ = *D*_*1*_*-D*_*2*_*-D*_*3*_*+D*_*4*_*+D*_*5*_*+D*_*6*_	
	λ_3_ = *-O*_*1*_*+O*_*2*_*+O*_*3*_*+rG*_*3*_	
E_8_(1,1,1)	λ_1_ = *G*_*3*_*-G*_*4*_	Condition 3: *G*_*3*_<*G*_*4*_、*D*_*1*_*+D*_*4*_*+D*_*5*_*+D*_*6*_*>D*_*2*_*+D*_*3*_、*O*_*1*_<*O*_*2*_+*O*_*3*_+ *rG*_*3*_
	λ_2_ = *-D*_*1*_*+D*_*2*_*+D*_*3*_*-D*_*4*_*-D*_*5*_*-D*_*6*_	
	λ_3_ = *O*_*1*_*-O*_*2*_*-O*_*3*_*-rG*_*3*_	

The analysis in the paragraph above demonstrates how decisions are influenced by the government, developers, and purchasers. The incremental cost and sales risk of prefabricated buildings must be kept low if the government wishes to encourage developers to develop prefabricated buildings.

From equilibrium point E_2_, it is clear that the government and developers do not have much enthusiasm for prefabricated buildings. But it will promote the growth of purchaser demand for prefabricated buildings when purchasers realize that the benefits brought by prefabricated buildings can cover the purchase cost of more expenditure. As can be shown, market demand can be leveraged to increase the supply of prefabricated buildings in locations where purchasers have a high level of awareness of them, fostering the enthusiasm of developers to develop prefabricated buildings.

However, in developing countries, prefabricated building development is frequently delayed, and purchaser acceptance is low. In addition, the environmental problems caused by the government′s lack of economic incentives for promoting prefabricated buildings are mainly concentrated in water pollution, carbon emissions, and construction waste. Construction-related industries are the main source of global emissions, accounting for 50% of air pollution and 42% of global greenhouse gas emissions[1]. Liu et al. (2015) calculated that the use of prefabricated buildings could reduce water consumption by about 20%, carbon emissions by about 7%, and garbage emissions by about 22% [63]. Li et al. (2018) calculated that prefabricated buildings could reduce about 80% of construction waste by taking rural prefabricated buildings as an example [64]. Based on this, it is known that the environmental governance cost caused by the non-implementation of prefabricated buildings is huge, and the negative impact of environmental damage is long-term and difficult to repair. Therefore, *G*_*4*_ is considered to be larger here. Moreover, the amount of subsidy expenditure is quite different between the governments of developing countries and those of developed countries. Therefore, *G*_*3*_ is the key factor for the difference between the two. Given the limited government spending in developing countries, it should be considered that the government′s economic subsidy is not very large. Therefore, *G*_*3*_ is considered smaller here. The following part mainly analyzes the evolution process when *G*_*3*_<*G*_*4*_.

From equilibrium point E_5_, it can be seen that ignoring the benefits of the purchasers will lead to a shrinking demand for prefabricated buildings. It will lessen developers’ enthusiasm to develop prefabricated buildings and could make the government’s subsidy incentives formal and useless. In this case, the direction of the stable evolution of the three parties lies in the choice of the purchasers. Therefore, it is crucial for developing countries to pay attention to the benefits of purchasers buying prefabricated buildings in order to ensure the effective promotion of prefabricated buildings.

According to the above analysis, in order to make it evolve to point E_8_, it needs to meet condition 3. Combined with the hypothesis in this paper, it can be seen that condition 3 means that the inequality *G*_*3*_<*G*_*4*_、*O*_*1*_<*O*_*2*_+*O*_*3*_+ *rG*_*3*_ is satisfied at the same time, that is, *G*_*3*_<*G*_*4*_、*rG*_*3*_> *O*_*1*_-*O*_*2*_-*O*_*3*_ is established at the same time. Among them, *rG*_*3*_ represents the portion of the subsidy for the purchasers. *O*_*1*_-*O*_*2*_-*O*_*3*_ represents the difference between the incremental cost of the purchasers’ purchase of prefabricated buildings and the benefits such as policies and the residential environment. The difference is regarded as the algebraic sum of other benefits except government subsidies obtained by the purchasers to purchase prefabricated buildings. Based on this, Condition 3 indicates that the government’s reasonable subsidy should be lower than the environmental governance cost paid by the government for not promoting prefabricated buildings. And the portion of the subsidy for the purchasers should be higher than the algebraic sum of other benefits obtained by the purchasers to purchase the prefabricated buildings.

In summary, the reasonable range of government subsidies can be expressed as inequality (12).


$$\frac{O_{1}-O_{2}-O_{3}}{r}<G_{3}<G_{4}$$ (12)

## Simulation analysis

In order to show the game evolution process of the behavior strategy of the three parties more intuitively, the simulation method is used to explore the influence law of model parameters on the evolutionary game process of the three parties. Here, we mainly analyze the sensitivity of relevant parameters when they evolve to point E_8_. After analysis, the initial simulation parameters are determined as follows: *G*_*3*_ = 200, *G*_*4*_ = 400, *D*_*1*_ = 200, *D*_*2*_ = 100, *D*_*3*_ = 300, *D*_*4*_ = 100, *D*_*5*_ = 100, *D*_*6*_ = 100, *O*_*1*_ = 200, *O*_*2*_ = 150, *O*_*3*_ = 150, *r* = 0.1. Parameters satisfy *G*_*3*_*<G*_*4*_, *D*_*1*_+*D*_*4*_+*D*_*5*_+*D*_*6*_>*D*_*2*_+*D*_*3*_, *O*_*1*_<*O*_*2*_+*O*_*3*_+*rG*_*3*_, to ensure that the evolution results reach E_8_(1,1,1).

1. Impact analysis of strategic probability

**Fig 5:**
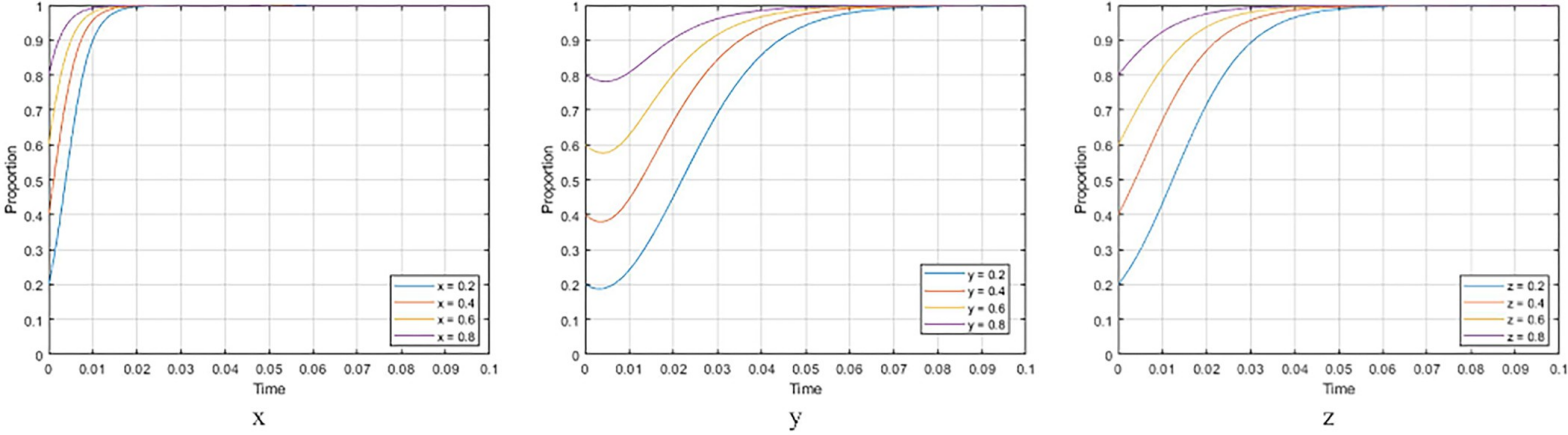
*x*, *y*, *z* Evolution curves with different values.

Fig 5 shows the evolution paths of *x*, *y*, and *z* with different values. It can be seen that no matter how likely the three parties choose (Incentive, Develop prefabricated buildings, Purchase prefabricated buildings), the three parties will eventually achieve interest equilibrium. However, the purchasers′ choice has the greatest impact on the realization process of the tripartite interest balance. Promoting purchasers′ active choice of prefabricated buildings through market guidance is a crucial strategy for increasing the degree of prefabricated buildings promotion.

2. Analysis of the influence of government incentive degrees

**Fig 6:**
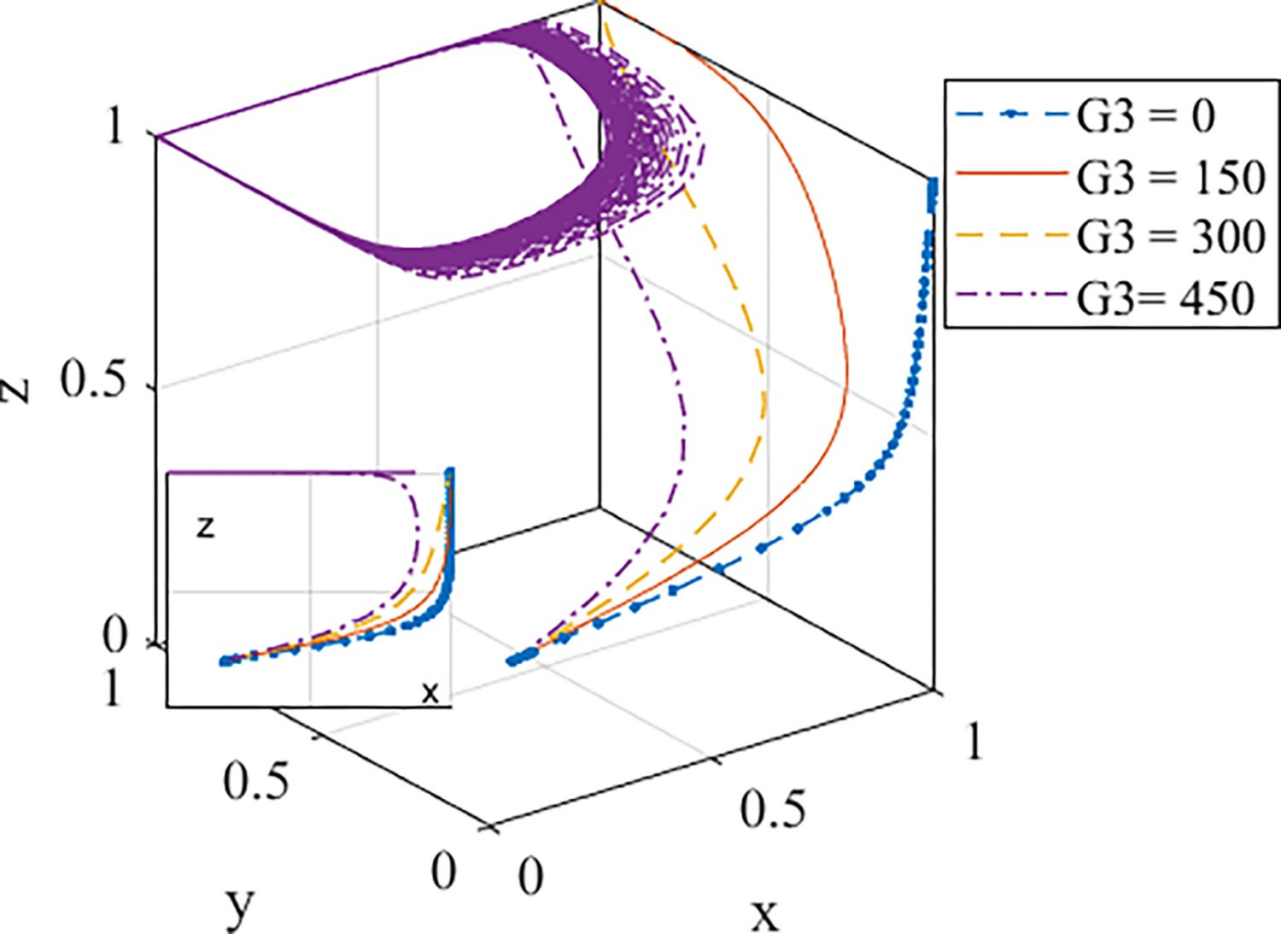
Evolution path diagram of the game with the change of *G*_*3*_.

*G*_*3*_ represents the economic subsidies paid by the government, including direct subsidy costs, tax deductions, and incentives, and the values of *G*_*3*_ are 0, 150, 300, and 450, respectively. The evolution path is shown in Fig 6. If there is no economic incentive, namely, *G*_*3*_ = 0, the curve will rapidly evolve to the point (1,0,1). This demonstrates that the government cannot reach an ideal stable state without using economic incentives to subsidize developers. When the government adopts subsidies, the rate of evolution is accelerated by the size of the subsidy expenditure. The evolution process is relatively slow in the early stage and fast in the middle stage, which indicates that the pressure of government subsidies is large in the early stage. With the continuous development and expansion of the market, the incremental cost of prefabricated buildings is reduced by industry development, and the pressure of government subsidies is reduced. Therefore, the evolution rate is accelerated. In addition, if the subsidy exceeds the cost of environmental governance, that is, when *G*_*3*_ = 450, it indicates that *G*_*3*_ exceeds the bearing range of developing countries and cannot reach a stable state at this time. Therefore, it can be seen that it is essential for developing countries to adopt a reasonable subsidy amount.

3. Analysis of the impact of purchaser interest

**Fig 7:**
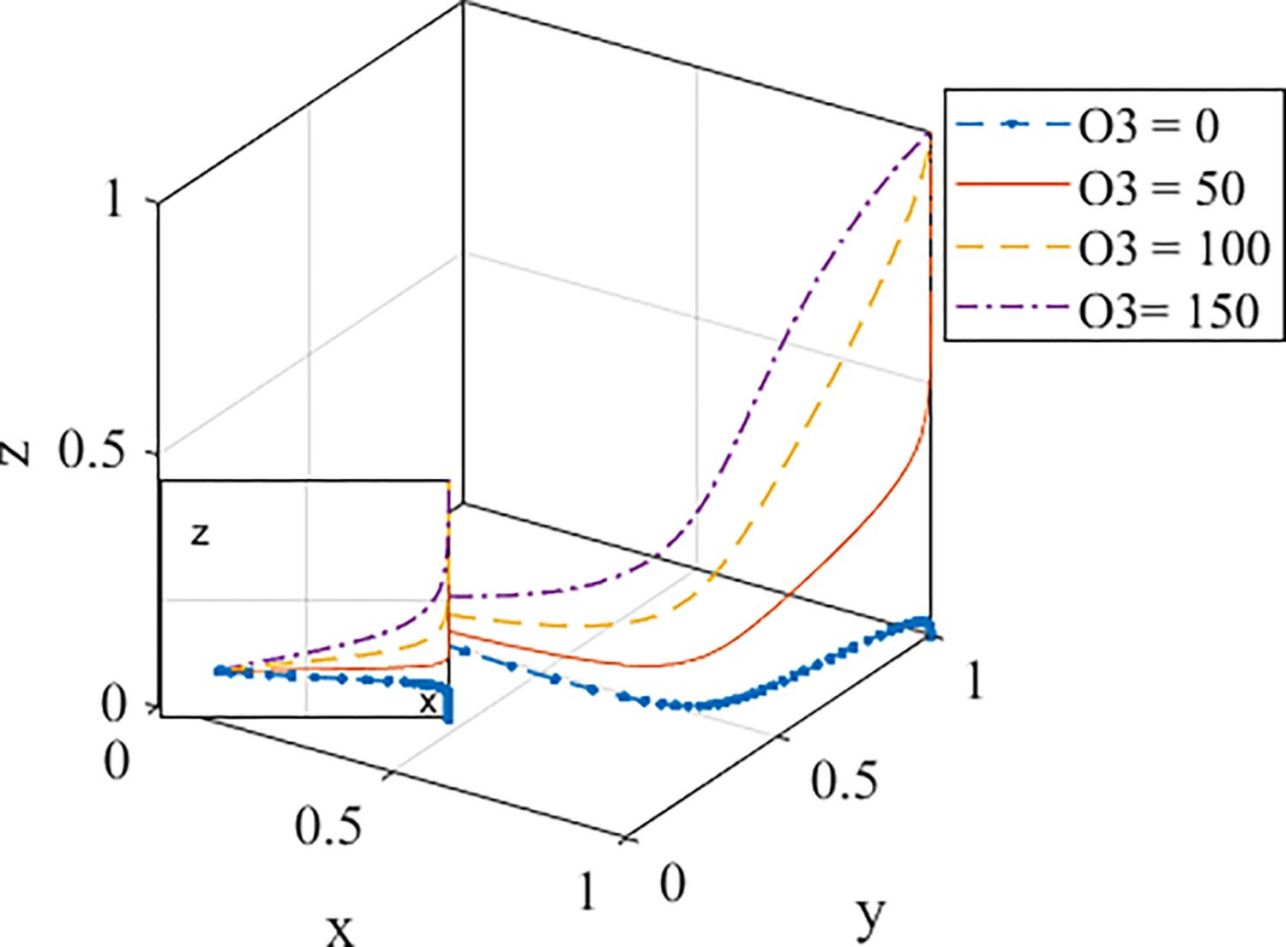
Evolution path diagram of the game with the change of *O*_*3*_.

*O*_*3*_ represents the benefits of the residential environment obtained by purchasers of prefabricated buildings, and the values of *O*_*3*_ are 0, 50, 100, and 150, respectively. The evolution path is shown in Fig 7. When the fabricated buildings have no residential environmental advantage compared with the cast-in-situ buildings, namely, *O*_*3*_ = 0, the curve will rapidly evolve to the point (1,1,0). This demonstrates that if the purchasers buy fabricated buildings without residential environmental benefits, they cannot achieve the ideal tripartite stability. At this time, the promotion of prefabricated buildings is not accepted by purchasers, and the weak demand will seriously limit the sustainable development of prefabricated buildings. Therefore, the residential environmental benefit *O*_*3*_ is the key parameter affecting the promotion effect of prefabricated buildings. The better the residential environment of prefabricated buildings, the faster the evolution rate of purchasers′ choice of prefabricated buildings. This shows that if the government improves the residential environment standards of prefabricated buildings, it can drive market demand for prefabricated buildings and further enhance the promotion of prefabricated buildings.

4. Developers′ initiative analysis

**Fig 8:**
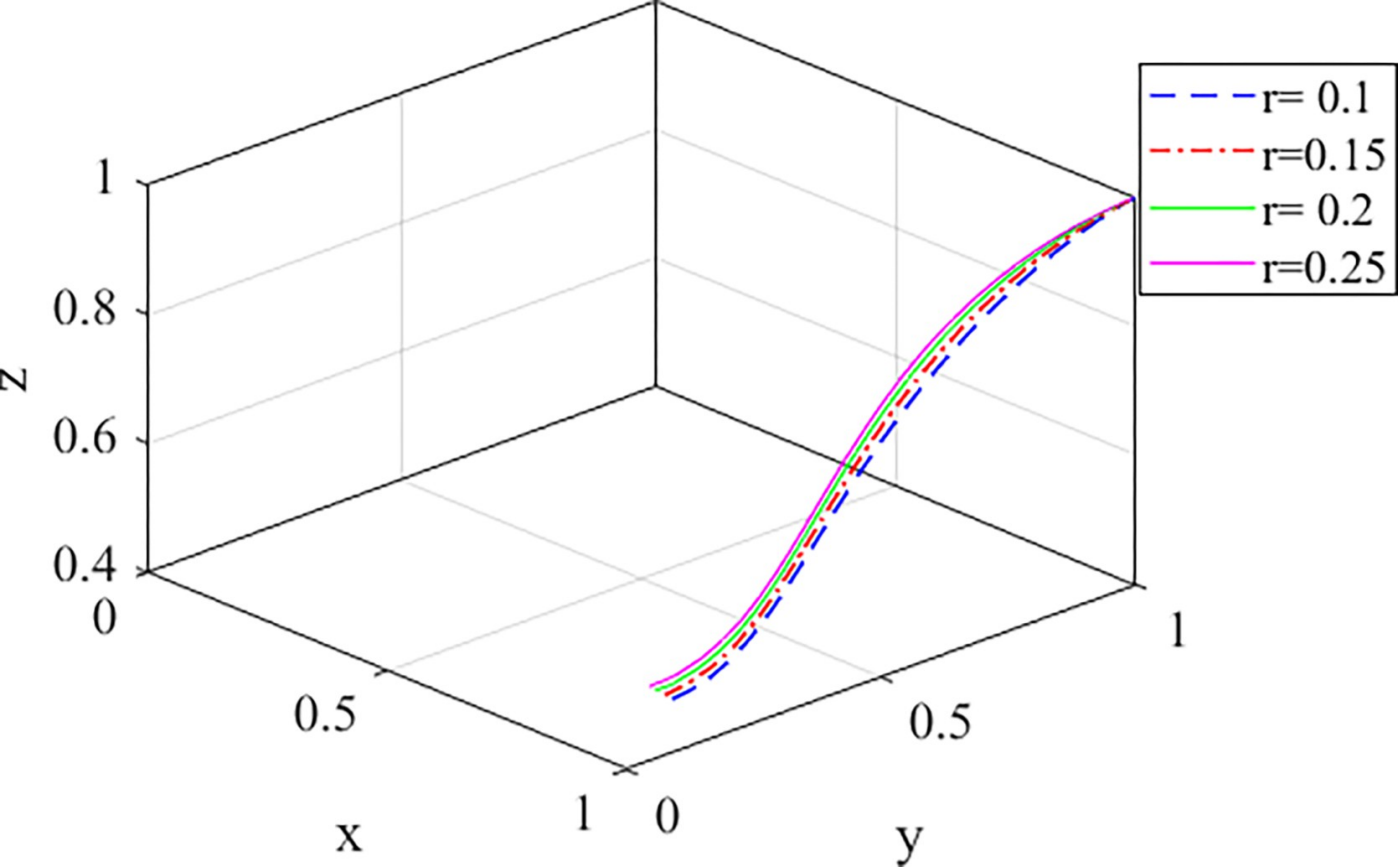
Evolution path diagram of the game with the change of *r*.

*r* represents the proportion of subsidies received by the purchasers in the total subsidy amount. The values of *r* are 0.1, 0.15, 0.2, and 0.25, respectively. The evolution path is shown in Fig 8. The government prefers incentives, obviously, because it needs to provide subsidies to ensure that developers are enthusiastic about choosing prefabricated buildings. However, as *r* increases, this tendency will weaken. For developers, increasing the proportion of the subsidy for the purchasers *r* will reduce the enthusiasm of developers to choose prefabricated buildings and slow down the evolution rate. Therefore, the government should focus on improving the residential environmental benefits of prefabricated buildings to promote the evolution rate of purchasers and reduce *r* to ensure the enthusiasm of developers.

## Discussion and limitations

### Discussion

1. Market demand can effectively stimulate the supply of prefabricated buildings. In the existing research on promoting prefabricated buildings, scholars ’ analysis of the demand side is relatively simple, and the current policies pay less attention to purchasers. However, based on the stability point E_5_, it can be seen that the decision-making of developers is affected by the sales risk, and the preference of purchasers for prefabricated buildings determines the size of the risk of their sale. Therefore, market demand is a crucial factor restricting prefabricated buildings. Based on the stability point E_2_, the willingness to demand is still strong when the purchasers believe that the income from purchasing prefabricated buildings is higher than the expenditure. Therefore, there is still a lot of room for improvement on the demand side of the prefabricated building market. Purchasers′ acceptance of prefabricated buildings can be increased to stimulate market demand. This will increase developers’ enthusiasm for promoting prefabricated buildings.
2. Government subsidies are a common factor affecting the enthusiasm of the government, developers, and purchasers. Based on the simulation results, it can be seen that the government’s financial pressure limits the amount of government subsidies. When the amount of subsidies exceeds the cost of environmental governance, it will decrease the government’s enthusiasm to promote. However, most of the existing research lacks the consideration of government financial pressure when analyzing subsidy expenditure, which leads to the limitation of the practical applicability of the analysis conclusion. In addition, the amount of subsidies is also a part of the income of developers and purchasers. Therefore, to effectively promote the enthusiasm of all parties under limited subsidies and determine a reasonable scope of subsidies must consider the interests of the three parties. Based on this, the government’s reasonable subsidy should be lower than the environmental governance cost paid by the government for not promoting prefabricated buildings. And the portion of the subsidy for the purchasers should be higher than the algebraic sum of other benefits obtained by the purchasers who purchase the prefabricated buildings.
3. The residential environment can effectively enhance the immediate interests of purchasers. The existing research on purchasers is relatively simple, and the key factors to protect the interests of purchasers have not been clearly put forward. Measures with high subsidies often ignore the government’s financial pressure and have low applicability. In addition, high subsidies may also lead to higher purchase prices. Combined with the simulation analysis, it can be seen that the government should choose a lower proportion of the subsidy to give to the purchasers. This can ensure that in the case of limited expenditure, sufficient subsidies can be given to developers in the early stages. Through the above simulation analysis, it can be seen that the residential environment index *O*_*3*_ is the key parameter to determining whether the promotion of prefabricated buildings can evolve into an ideal state. A good residential environment promotes purchasers′ willingness to buy prefabricated buildings. If the government fails to effectively establish standards and mechanisms related to prefabricated buildings and their benefits for residential environments, the long-term weakness of the demand side will seriously limit the sustainable development of prefabricated buildings. Therefore, the government should formulate a completely green building standard system for prefabricated buildings so as to restrict developers from paying attention to the residential environmental benefits of prefabricated buildings. In addition, the government should improve the related technologies and mechanisms for the residential environmental benefits of prefabricated buildings. This will ensure that the purchasers can get the ideal residential environment. The measures outlined above can also reduce the sales risk of prefabricated buildings and improve their promotion effects. It is conducive to improving purchasers’ preference for prefabricated buildings, thus reducing the sales risk of developing them and the requirements of government financial expenditure.

### Limitations

Given that the demand for prefabricated buildings in developing countries is relatively low and the development is not yet mature, this study constructs a tripartite game model based on the government, developers, and purchasers. Further, it reveals the coupling mechanism of tripartite cooperation.

In the subject selection, this study comprehensively discusses the main interest representatives of the supply and demand process of prefabricated buildings by summarizing and comparing the research results of scholars in various places. However, when developers make decisions, they face the game between prefabricated manufacturers and construction parties. To further describe the relationship between their decision-making process, they need to be realized through four-party games or even five-party games.

In the process of parameter index selection, this study further deepens the careful consideration of the three-party interest parameters based on existing research. However, due to the wide range of parameter indicators, it is necessary to further refine them to accurately describe the influencing factors of the main decision-making process.

In addition, government subsidies are an important parameter for implementing the strategy. The results finally point out the scope of reasonable government subsidies, but the financial status and willingness to promote different countries differ. From the perspective of this study, government subsidies are indispensable. It does not apply to this result for countries that cannot afford the subsidy amount.

Although this study has some limitations, the viewpoints and conclusions still have strong practical significance for developing countries that want to further promote prefabricated buildings.

## Conclusions

It is critical to encourage the development of prefabricated buildings in order to promote energy conservation and emission reduction, environmental protection, and related scientific and technological progress. In order to solve the current problems of the slow development of prefabricated buildings and their low acceptance by purchasers. Based on the tripartite evolutionary game of government-developers-purchasers, this paper discusses the limiting factors that affect the further promotion of prefabricated buildings and analyzes the sensitivity of key parameters to subject behavior decision-making by simulation.

Based on the above research, the following conclusions are drawn:

1. The lack of government subsidies or the weak demand of the purchasers for prefabricated buildings is an important factor in determining whether the government, developers, and purchasers can cooperate in promoting prefabricated buildings. Exploring the reasonable scope of government subsidies and improving purchasers′ acceptance of prefabricated buildings has become an important way to promote the promotion of prefabricated buildings.
2. The government’s reasonable subsidy should be lower than the environmental governance cost paid by the government for not promoting prefabricated buildings. And the portion of the subsidy for the purchasers should be higher than the algebraic sum of other benefits obtained by the purchasers to purchase the prefabricated buildings. And the proportion of government subsidies for the purchasers should be small to ensure that developers have sufficient enthusiasm under limited expenditure.
3. The vested interests of the purchasers can be greatly benefited by improving the residential environment of prefabricated buildings. It can be seen that the promotion of prefabricated buildings can be promoted through the stimulation of the demand side of prefabricated buildings. If the government establishes standards and mechanisms related to the residential environmental benefits of prefabricated buildings, it can not only reflect the people-oriented style but also smoothly promote the development of the prefabricated building market.

## Supporting information

S1 Code Code 3. (TXT)
